# Electron-event representation data enable efficient cryoEM file storage with full preservation of spatial and temporal resolution

**DOI:** 10.1107/S205225252000929X

**Published:** 2020-08-07

**Authors:** Hui Guo, Erik Franken, Yuchen Deng, Samir Benlekbir, Garbi Singla Lezcano, Bart Janssen, Lingbo Yu, Zev A. Ripstein, Yong Zi Tan, John L. Rubinstein

**Affiliations:** a Molecular Medicine Program, The Hospital for Sick Children, 686 Bay Street, Toronto, Ontario M5G 0A4, Canada; b Department of Medical Biophysics, The University of Toronto, 101 College Street, Toronto, Ontario M5G 1L7, Canada; c Thermo Fisher Scientific, Achtseweg Noord 5, 5651 GG Eindhoven, The Netherlands; d Department of Biochemistry, The University of Toronto, 1 King’s College Circle, Toronto, Ontario M5S 1A8, Canada

**Keywords:** electron-event representation, cryoEM, direct detector device

## Abstract

Direct detector device (DDD) cameras have revolutionized electron cryomicroscopy (cryoEM) with their high detective quantum efficiency (DQE) and output of movie data. A high ratio of camera frame rate (frames per second) to camera exposure rate (electrons per pixel per second) allows electron counting, which further improves the DQE and enables the recording of super-resolution information. Movie output also allows the correction of specimen movement and compensation for radiation damage. However, these movies come at the cost of producing large volumes of data. It is common practice to sum groups of successive camera frames to reduce the final frame rate, and therefore the file size, to one suitable for storage and image processing. This reduction in the temporal resolution of the camera requires decisions to be made during data acquisition that may result in the loss of information that could have been advantageous during image analysis. Here, experimental analysis of a new electron-event representation (EER) data format for electron-counting DDD movies is presented, which is enabled by new hardware developed by Thermo Fisher Scientific for their Falcon DDD cameras. This format enables the recording of DDD movies at the raw camera frame rate without sacrificing either spatial or temporal resolution. Experimental data demonstrate that the method retains super-resolution information and allows the correction of specimen movement at the physical frame rate of the camera while maintaining manageable file sizes. The EER format will enable the development of new methods that can utilize the full spatial and temporal resolution of DDD cameras.

## Abstract 2

Electron-event representation is a new data format for cryoEM that preserves the full temporal and spatial resolution of movies from direct detector device cameras.

## 1. Introduction   

**Figure 1:**
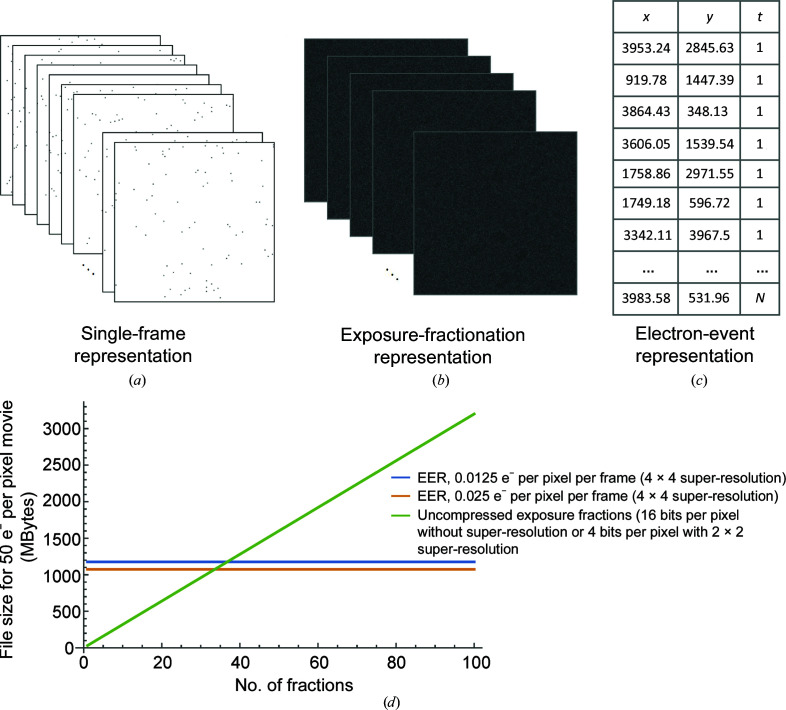
The EER file format. (*a*) Direct detector device (DDD) cameras operating in counting mode record the impact positions of electrons on the sensor at the frame rate of the camera. (*b*) Conventionally, groups of successive movie frames are summed to fractionate the exposure, reducing the size of movie files from DDD cameras. This exposure fractionation requires decisions to be made by the experimentalist about the temporal resolution to be preserved in order to avoid loss of information from specimen movement during imaging. (*c*) The electron-event representation (EER) file format uses efficient data encoding, marking the position and time (in raw frame number) for each electron. (*d*) Example data sizes under typical conditions. All reported data sizes assume a total exposure on the specimen of 50 e^−^ Å^−2^, a pixel size of 1 Å, a frame size of 4096 × 4096 pixels and neglect any loss of electrons between specimen exposure and detection with the camera. Green curve: data size for uncompressed exposure fractions with 16 bits per pixel or (equivalently) four bits per pixel with 2 × 2 super-resolution. Blue and orange curves: EER file sizes with 4 × 4 super-resolution at exposure rates of 0.0125 and 0.025 e^−^ Å^−2^ per frame, respectively. The EER file size depends only on the total electron exposure and the exposure rate of the camera, while the file size for conventional movies depends on the number of fractions recorded. EER thus preserves the full temporal resolution of the electron-detection events and requires a smaller file size for many practical fractionation conditions. More camera frames are required to reach the same total exposure when a lower exposure rate is used, and consequently EER files with 0.0125 e^−^ Å^−2^ per frame are larger than those with 0.025 e^−^ Å^−2^ per frame, as described in (5).

Complementary metal-oxide semiconductor (CMOS) direct detector device (DDD) cameras for cryoEM provide improved detective quantum efficiency (DQE) compared with other detectors (McMullan *et al.*, 2016 ▸). Furthermore, these cameras can record movies of the specimen during irradiation. Movies are output from the detector as raw ‘camera frames’ [Fig. 1 ▸(*a*)], with successive frames summed to produce ‘exposure fractions’ that are saved for image processing [Fig. 1 ▸(*b*)]. Movie output has three advantages (Li *et al.*, 2013 ▸; Campbell *et al.*, 2012 ▸). Firstly, it facilitates further improvement of the DQE through the implementation of electron counting, where an algorithm is used to detect, localize and normalize the signal from each electron in individual camera frames. Secondly, it allows super-resolution imaging by recording the positions of electrons with an accuracy finer than the size of the physical pixels of the sensor. Finally, DDD movies make it possible to account for radiation damage to the specimen and correct the beam-induced specimen motion and microscope-stage drift that occur during imaging. The DQE is improved by electron counting because the signal contributed to the image by each electron varies stochastically (McMullan, Faruqi *et al.*, 2009 ▸) and consequently counting electrons normalizes this signal (Li *et al.*, 2013 ▸). For electron counting, the exposure per frame is limited to one electron for every ∼40–100 pixels. This low density of electrons per frame allows individual electrons to be detected with a low probability of two electrons impinging on the same region during the recording of the frame, which would lead to the undercounting of electrons in a phenomenon known as ‘coincidence loss’. Each electron deposits energy into multiple pixels upon hitting the sensor, and consequently the centre of the impact event can be localized to a specific region of a pixel in order to allow super-resolution imaging (Li *et al.*, 2013 ▸). Recording super-resolution information also improves the DQE of the camera within the physical Nyquist frequency by reducing aliasing (McMullan, Chen *et al.*, 2009 ▸).

Beam-induced motion and specimen drift, which blur the images of ice-embedded protein complexes in integrated exposures, can limit the resolution attainable by cryoEM. Numerous schemes have now been implemented to correct this motion (Ripstein & Rubinstein, 2016 ▸). Some approaches treat the image on the entire area of the detector as moving in unison (Li *et al.*, 2013 ▸; Grant & Grigorieff, 2015 ▸). Others divide the detector into patches (Zheng *et al.*, 2017 ▸) or work on individual particle images, using either the shift-dependent average of exposure fractions (Rubinstein & Brubaker, 2015 ▸) or a projection of a 3D map (Zivanov *et al.*, 2019 ▸; Bai *et al.*, 2013 ▸; Scheres, 2014 ▸; Brilot *et al.*, 2012 ▸; Campbell *et al.*, 2012 ▸) to guide alignment. Finally, radiation damage to specimens means that the early part of each exposure contains more high-resolution information than the later part, and this loss of information can be accounted for when averaging exposure fractions (Baker *et al.*, 2010 ▸; Rubinstein & Brubaker, 2015 ▸; Feng *et al.*, 2017 ▸; Grant & Grigorieff, 2015 ▸) or during 3D reconstruction (Scheres, 2014 ▸; Zivanov *et al.*, 2019 ▸).

The smallest possible exposure fraction from a camera is a single camera frame, with current hardware frame rates for ∼4k × 4k pixel sensors of between 40 and 1500 frames per second. Consequently, camera movie modes have the potential to produce enormous volumes of data. For example, a 4096 × 4096 pixel sensor with a readout rate of 400 frames per second and with pixel values stored as four bits of information would produce 3.125 GiB of information each second. Movies must be recorded over multiple seconds for electron counting with an appropriate total electron exposure and magnification for 2–3 Å resolution reconstructions of a biological specimen (Ripstein & Rubinstein, 2016 ▸). Therefore, while DDDs have revolutionized cryoEM and structural biology as a whole, they have placed great demands on current computational data-storage infrastructure. Because storing the entirety of these movies is not usually practical, experimentalists must make decisions not just about magnification (Å per pixel), total electron exposure on the sample (e^−^ Å^−2^) and camera exposure rate (e^−^ per pixel per second), but also about how to best fractionate the exposures by summing successive frames after electron counting. If exposures are fractionated too finely, the file sizes can be excessively large. If exposures are fractionated too coarsely, significant motion can occur within one fraction, compromising the resolution of the 3D structures that can be calculated from the data. These decisions are made at the time of data collection and the microscopist runs the risk of realizing during analysis that their data-acquisition strategy was not optimal.

In this paper, we describe electron-event representation (EER), an image-recording strategy developed at Thermo Fisher Scientific for their Falcon cameras. We show that storing EER data removes the need to decide on an exposure-fractionation strategy during imaging, enabling the optimal correction of specimen motion. In addition, we demonstrate that EER files record super-resolution information in images, allowing 3D reconstruction beyond the Nyquist frequency.

## 2. Methods   

### 2.1. Specimen preparation   

Human apoferritin was a gift from Ms Taylor Sicard and Professor Jean-Philippe Julien (The Hospital for Sick Children) and was used at 10 mg ml^−1^. Holey gold grids with a regular array of ∼2 µm holes were prepared as described previously (Marr *et al.*, 2014 ▸). The grids were subjected to 15 s of glow discharge in air before freezing in liquid ethane using a Gatan CP3 grid-freezing device. The grid-freezing device chamber was at room temperature and 90% relative humidity and blotting was performed for 10 s with an offset of −0.5 mm.

### 2.2. Data collection   

Images were acquired as described below with a Titan Krios G3 electron microscope from Thermo Fisher Scientific operating at 300 kV and equipped with a Falcon 3EC camera and a prototype EER module (used for intra-fraction motion-correction experiments) and later with a prototype Falcon 4 camera (used for super-resolution experiments). Automatic data collection was performed with the *EPU* software package. For EER intra-fraction motion correction, 325 movies of human light-chain apoferritin were collected with the Falcon 3EC camera at a 75 000× nominal magnification, corresponding to a calibrated pixel size of 1.06 Å. Falcon 3EC movies were recorded simultaneously in both EER format with 2312 raw frames per movie as well as 16-bit MRC format with 30 fractions per movie. The camera exposure rate and the total exposure of the specimen were 0.80 e^−^ per pixel per second and ∼41 e^−^ Å^−2^, respectively, with a defocus ranging from 0.4 to 1.6 µm. Following completion of this aspect of the work, we replaced the Falcon 3EC camera with a prototype Falcon 4 camera, which increased the physical frame rate from 40 to 250 frames per second. Consequently, for EER super-resolution data, 100 movies were collected on the same microscope but using the prototype Falcon 4 camera. A nominal magnification of 47 000× gave a calibrated pixel size of 1.64 Å. This camera did not allow simultaneous recording of EER data and conventional movies. After collection, these EER files could be converted to standard MRC files with the desired exposure fractionation. The camera exposure rate was 5 e^−^ per pixel per second and the total exposure on the specimen was ∼45 e^−^ Å^−2^. Movies were stored in EER format with 5782 raw frames per movie. The defocus in this data set ranged from 0.6 to 1.1 µm.

### 2.3. EER image handling   

The prototype EER module for the Falcon 3EC camera ran custom firmware with real-time EER encoding, streaming the data to a dedicated computer running the Ubuntu 16.04 operating system. With the Falcon 4 camera, the EER files were stored using the standard Falcon 4 storage infrastructure, which normally records MRC exposure-fractionation stacks. Electron-detection events were stored with run-length encoding as described below. Frames were packed into a BigTIFF-compliant file format with a gain-reference image stored separately in an MRC file. Information about defects was encoded in the same gain reference with a value of ‘0’. EER files were decoded using a hybrid CPU/GPU implementation of the decoding algorithm. To utilize subpixel information optimally for both super-resolution and non-super-resolution cases, all decoded images were reconstructed on the full 4 × 4 supersampled image grid and subsequently Fourier-cropped to the desired resolution. For single-particle cryoEM, EER files were converted to standard exposure-fractionated image stacks that could be used in a standard image-processing pipeline. In the final correction of motion for individual particle images, the EER files were decoded with the desired supersampling (*i.e.* 4 × 4 oversampling followed by Fourier cropping), image shifts were applied and exposure weighting was performed as described previously (Rubinstein & Brubaker, 2015 ▸). The application of image shifts to data from EER files was performed by placing electrons on shift-compensated positions rather than first composing an image and then applying shifts by interpolation in real space or phase changes in Fourier space. The procedure of shifting electron positions prior to image reconstruction is less expensive computationally than image interpolation and prevents image-interpolation artefacts. Efficient gain correction was performed by retrieving the gain-correction coefficient from the uncorrected pixel locations for each detected electron and applying it as a weighting factor for the contribution of the electron to its shifted position. During these procedures, the individual particle-motion trajectories were either smoothed with a cubic spline interpolation or not interpolated as a control, as described below.

### 2.4. Single-particle cryoEM image analysis   

For the Falcon 3EC data set, 325 16-bit MRC movies were imported into *cryoSPARC* v2 (Punjani *et al.*, 2017 ▸). Movie frames were aligned with an improved implementation of *alignframes_lmbfgs* (Rubinstein & Brubaker, 2015 ▸) within *cryoSPARC* v2 and contrast-transfer function (CTF) parameters were estimated from the average of aligned frames with *CTFFIND*4 (Rohou & Grigorieff, 2015 ▸). 335 137 particle images were selected and beam-induced motion for individual particles was corrected with an improved implementation of *alignparts_lmbfgs* (Rubinstein & Brubaker, 2015 ▸) within *cryoSPARC* v2. After two rounds of 2D classification, 291 408 particle images were selected and divided into three beam-tilt groups. Initial homogeneous refinement was performed in *cryoSPARC* v2 without CTF refinement. The alignment information in the *cryoSPARC* .cs file was converted to *RELION* 3.0 .star file format using the *pyem* package (https://10.5281/zenodo.3576630), allowing per-particle CTF and per-group beam tilt to be calculated in *RELION* 3.0. Refinement of CTF and beam-tilt parameters without alignment in *RELION* (Zivanov *et al.*, 2020 ▸) but with imposed octahedral symmetry produced a 3D reconstruction at 2.14 Å resolution. Super-resolution images of the particles with a new pixel size of 0.7067 Å were extracted with and without intra-fraction motion correction as described above. Refinement of the CTF and beam-tilt parameters was performed in *RELION* using the previously determined angles. An equivalent analysis was performed on the first six 0.70 e^−^ Å^−2^ fractions of the EER movies.

For super-resolution experiments with the Falcon 4 data set, 100 EER movies were decompressed and converted to 32-bit floating-point MRC format. Movie fractions were aligned by patch-based motion correction, and CTF parameters were determined with patch CTF estimation in *cryoSPARC* v2 (Punjani *et al.*, 2017 ▸). Templates for automatic particle selection were generated by 2D classification of manually selected particles. 247 312 single-particle images were selected from the aligned fractions, and beam-induced motion correction for individual particles and exposure weighting was performed in *cryoSPARC* v2 in the same way as described for the Falcon 3EC data set. A subset of 214 410 particle images was selected by 2D classification. Homogeneous refinement in *cryoSPARC* v2 with imposed octahedral symmetry, per-particle defocus refinement and higher-order aberration correction (Zivanov *et al.*, 2020 ▸), including beam tilt and trefoil aberration, yielded a map at 3.3 Å resolution. Super-resolution images of the same particles with a pixel size of 0.82 Å were extracted from EER movies with and without random subpixel electron placement as described above. Similar homogeneous refinement of the super-resolution particles with and without random subpixel electron placement yielded maps at 2.8 and 2.4 Å resolution, respectively.

## 3. Results   

### 3.1. Theoretical basis for EER   

Conventional representations of cryoEM movies store pixel intensities for each exposure fraction. In contrast, in EER each electron-detection event is recorded as a tuple of position and time (*x*, *y*, time), indicating where and when the electron was detected on the sensor [Fig. 1 ▸(*c*)]. As discussed earlier, owing to the need to avoid coincidence loss during electron counting, the number of detected electrons in a single camera frame must be ∼40–100 times smaller than the number of pixels in the frame. This inherent sparsity may be exploited for efficient encoding of pixel locations for the detected electrons. Assuming that in a single electron-counted camera frame each pixel is either not hit (value 0) or hit (value 1) by an electron, the stream of camera frame pixels can be modelled as a Bernoulli process with the probability *p* of an individual pixel being hit by an electron given by
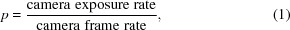
where the camera exposure rate has dimensions of e^−^ per pixel per second and the frame rate has dimensions of frames per second. The Shannon entropy (Shannon, 1948 ▸), *H*, of this Bernoulli process is
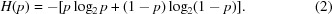
This Shannon entropy gives a lower bound on the number of bits per pixel needed to encode all events in a counted frame. Reaching this lower bound requires that the statistical model matches the statistics of the data and that an optimal data-compression scheme is used. A value of *p* ≠ 0.5 leads to *H*(*p*) < 1 and indicates that the camera frames can be compressed further. Recording electron locations on the sensor with super-resolution accuracy by subdivision of the physical pixels into *u* × *u* subpixels requires 2 log_2_(*u*) additional bits per electron. Consequently, the size in bytes, *D*, of an optimally compressed EER movie frame is given by
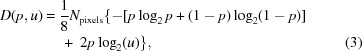
where *N*_pixels_ is the number of physical pixels in the sensor. For example, on a sensor with 4096 × 4096 pixels running at a frame rate of 240 frames per second, a camera exposure rate of 3 e^−^ per pixel per second gives *p* = 3/240 = 0.0125. When each pixel is subdivided into 4 × 4 subpixels (*u* = 4), an optimally compressed EER movie requires 301 kB per frame. Without recording super-resolution location information (*u* = 1) the same EER movie would require 199 kB per frame. The expected total size *S*_opt_ of an optimally compressed EER movie in bytes, neglecting any file-header information, is therefore given by
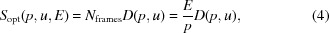
where *E* is the total electron exposure in the movie in e^−^ per pixel and *N*_frames_ is the number of camera frames recorded.

The EER format implemented for Falcon cameras uses run-length encoding (RLE) to reduce the data size. For each camera frame, the pixel distances between detected electrons, in the scanline order in which they are stored in memory, are encoded with a constant word length, *b*_RLE_. In the current algorithm, *b*_RLE_ was set at seven bits. The maximum value, *m*, for the given number of bits (*i.e.* *m* = 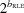 − 1 = 127 for *b*_RLE_ = 7 bits) is used to indicate that there was no electron detected after this maximum number of *m* pixels. This scheme does not achieve the optimal data compression and file size described in (4), but has the advantage of straightforward image encoding and decoding. The approximate total file size with RLE compression, *S*_RLE_, is given by the product of the total electron exposure *E*, the number of pixels *N*_pixels_ and the number of bits per electron *b*_RLE_ + 2 log_2_(*u*), but with a correction to account for the extra bits needed to represent the situation where no electron was detected after *m* pixels,
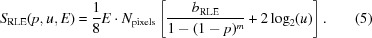
The optimal choice for *b*_RLE_ to minimize the file size depends on *p*. The use of seven bits enables small file sizes when typical exposure rates for electron counting are used. As justified below, the EER format implemented for Falcon cameras uses *u* = 4, meaning that the physical pixels are divided into 4 × 4 subpixels.

Fig. 1 ▸(*d*) shows typical EER file sizes (50 e^−^ per pixel total exposure with 1 Å per pixel) compared with standard uncompressed image formats such as MRC image-stack files (Cheng *et al.*, 2015 ▸). In contrast to the EER files, the MRC files described in the figure have reduced temporal resolution owing to averaging of successive frames. Where the example MRC files preserve super-resolution information, they use 2 × 2, rather than 4 × 4, subpixels. When more than ∼35 exposure fractions are recorded, EER files are smaller than uncompressed 16-bit MRC files or four-bit MRC files with 2 × 2 super-resolution information.

The intersection of the EER curve with the conventional fractionation approach curve will occur at a larger number of exposure fractions if a compressed image format is used, such as LZW–TIFF (Welch, 1984 ▸). However, the amount of image compression that can be achieved depends strongly on the image content and consequently it is difficult to compare these methods analytically. Electron counting can produce exposure fractions with pixel intensities represented by small integers encoded with as few as four bits per pixel. This type of image may be compressed efficiently. However, gain correction converts integer-valued pixels into real-valued pixels that must be represented by floating-point numbers or larger integers (for example 16 bits), producing files that do not compress efficiently. Similarly, the Fourier cropping of images to reduce file sizes while retaining the anti-aliasing benefits of super-resolution (McMullan, Chen *et al.*, 2009 ▸) requires pixel intensities to be represented by floating-point numbers or large integers, reducing the efficiency of file compression. The standard output from Falcon cameras includes both gain correction and real-space anti-aliasing and consequently these files do not compress efficiently. A current approach to image handling from other cameras is to store LZW–TIFF-compressed four-bit super-resolution images, applying the gain reference and performing Fourier cropping after decompression (Eng *et al.*, 2019 ▸). This approach reduces the file sizes for exposure fractions substantially compared with the uncompressed exposure fractions shown in Fig. 1 ▸(*d*). However, when used to preserve the full temporal and spatial resolution of movies, experiments indicate that LZW–TIFF files are approximately four times larger than the equivalent EER files and will not benefit from the streamlined file handling described below.

In principle, conventional movies saved with each exposure fraction consisting of a single super-resolution camera frame could subsequently be converted to EER format. However, the real-time output of EER data from the camera avoids saving extremely large uncompressed intermediate files even temporarily, which would make workflows prohibitively complicated. Lossy compression approaches have also been shown to reduce file sizes when the complete preservation of information is not required (Eng *et al.*, 2019 ▸). Consequently, conventional files that are smaller than the EER format can be produced, but doing so requires sacrificing temporal or spatial resolution.

### 3.2. Super-resolution imaging   

**Figure 2:**
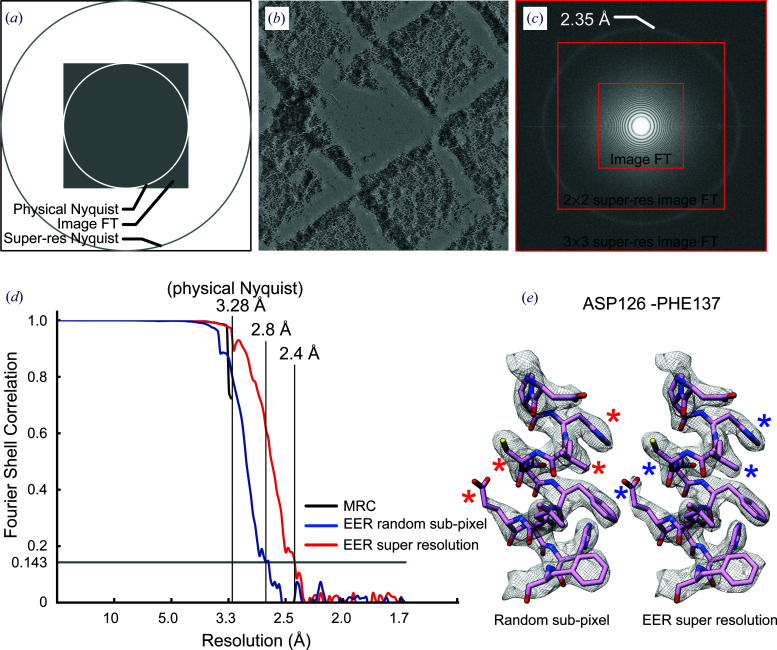
Super-resolution 3D reconstruction with EER files. (*a*) Illustration of the physical Nyquist frequency, information in square Fourier transforms beyond the physical Nyquist and the new Nyquist frequency from 2 × 2 supersampling of physical pixels. (*b*) Image of a cross-grating with polycrystalline gold recorded as an EER file. (*c*) Power spectrum from the image in (*b*), showing the image Fourier transform without super-resolution information (small red box), Fourier transform with 2 × 2 supersampling of physical pixels (medium red box) and 3 × 3 supersampling of physical pixels (large red box). (*d*) FSC curves from maps of human light-chain apoferritin with a physical Nyquist resolution of 3.28 Å: standard images (black curve), 2 × 2 supersampled with random subpixel electron placement (blue curve) and 2 × 2 supersampled with subpixel electron placement from the EER file (red curve). (*e*) Part of an α-helix from a 3D map of human light-chain apoferritin at 2.8 Å resolution (FSC = 0.143) from random subpixel information (left) and at 2.4 Å resolution (right) with super-resolution information from EER data. Asterisks (*) indicate features that are better resolved on the right than on the left.

Modern DDD cameras such as the Gatan K2 or K3, Direct Electron DE-16 or DE-64 and Thermo Fisher Scientific Falcon 3EC or 4 localize electrons with subpixel accuracy using a centroiding procedure before electron positions are recorded. As described above, this super-resolution information is preserved in the EER format by subdividing each physical pixel into *u* × *u* subpixels. Because the Nyquist resolution of a camera is given by two times the edge length of a pixel, the subdivision of physical pixels by a factor of *u* extends the Nyquist resolution by 1/*u*. Even without subpixel localization of electrons, images retain information beyond the Nyquist frequency because the corners of Fourier transforms encode spatial frequencies that are finer than the Nyquist frequency in the *x* or *y* direction of the image [Fig. 2 ▸(*a*)].

We investigated the ability of a Titan Krios electron microscope with a Falcon 4 camera and EER capability to record information beyond the physical Nyquist frequency of the camera sensor. Images of a standard cross-grating with polycrystalline gold were recorded with a physical pixel size of 2.7 Å [Fig. 2 ▸(*b*)]. The power spectrum from this image shows diffraction corresponding to 2.35 Å, or 2.3× the Nyquist resolution of 5.4 Å [Fig. 2 ▸(*c*)]. Therefore, it is evident that the electron-counting algorithm combined with the EER data format enables the recording of information beyond the physical Nyquist limit of the camera. Further, the experiment shows that the modulation transfer function of the camera is non-negligible between 2× and 3× the Nyquist resolution. To avoid a decrease in the camera DQE by aliasing of signal past 2× the Nyquist resolution, the EER format uses 4 × 4 subpixels.

To test whether the super-resolution capability of EER files could be applied to biological specimens, we imaged human light-chain apoferritin particles with a calibrated physical pixel size of 1.64 Å and a physical pixel Nyquist resolution of 3.28 Å. Movies were recorded as EER data with a total exposure of ∼45 e^−^ Å^−2^ on the specimen and a camera exposure rate of 5 e^−^ per pixel per second. These movies were then converted to 30 MRC-format exposure fractions. 3D reconstruction from 214 410 particle images extracted from 100 movies with a conventional refinement workflow gave a resolution by Fourier shell correlation of 3.3 Å [Fig. 2 ▸(*d*), black curve]. It should be noted that 3D reconstructions with resolutions close to the Nyquist frequency can suffer from artefacts that limit the ability to resolve their highest resolution features. Next, the same EER files were converted to movies with 30 fractions but with a pixel size of 0.82 Å (Nyquist resolution of 1.64 Å). Electrons were placed on a pixel grid that was 4 × 4 supersampled from the physical pixel grid of the camera. Subpixel positions were either chosen randomly or using the EER information. Subsequently, the images were Fourier-cropped to give an effective 2 × 2 supersampling of the physical pixel grid. 3D reconstruction from these images following the same workflow used with the conventional image files gave 3D maps with resolutions of 2.8 Å for random subpixel placement [Fig. 2 ▸(*d*), blue curve] and 2.4 Å for placement with information from EER [Fig. 2 ▸(*d*), red curve]. The resolution from the randomized subpixel information, 2.8 Å, is notable because it goes beyond the physical Nyquist resolution of 3.28 Å. This effect is owing to information past the Nyquist resolution found in the corners of the Fourier transform of the image [Fig. 2 ▸(*a*)], although improved motion correction in the supersampled images may also improve the map. The resolution from the reconstruction that used subpixel information from the EER file was 2.4 Å, 29 bins in Fourier space beyond the physical Nyquist resolution and 14 bins in Fourier space beyond the randomized subpixel control. Numerous features in the maps indicate improved resolution where EER subpixel information was used [Fig. 2 ▸(*e*), right, blue asterisks] compared with where random information was used [Fig. 2 ▸(*e*), left, red asterisks].

### 3.3. Intra-fraction motion correction enabled by EER imaging   

The ability to fractionate exposures up to the physical frame rate of the camera, without needing to store the data as high-frame-rate movies, provides the possibility of improved measurement and correction of beam-induced motion. However, estimating motion from extremely large numbers of fractions can be problematic for the current generation of motion-measurement algorithms (Rubinstein & Brubaker, 2015 ▸; Zivanov *et al.*, 2019 ▸; Zheng *et al.*, 2017 ▸). These problems may arise owing to decreased signal in shorter exposure fractions, and the increased number of dimensions in the optimization problem embedded in estimating motion. Consequently, estimating particle motion from movies with many short exposure fractions is likely to require new algorithms and approaches. Alternatively, motion can be measured from a smaller number of fractions, with the trajectory subsequently interpolated or extrapolated to the raw camera frames.

**Figure 3:**
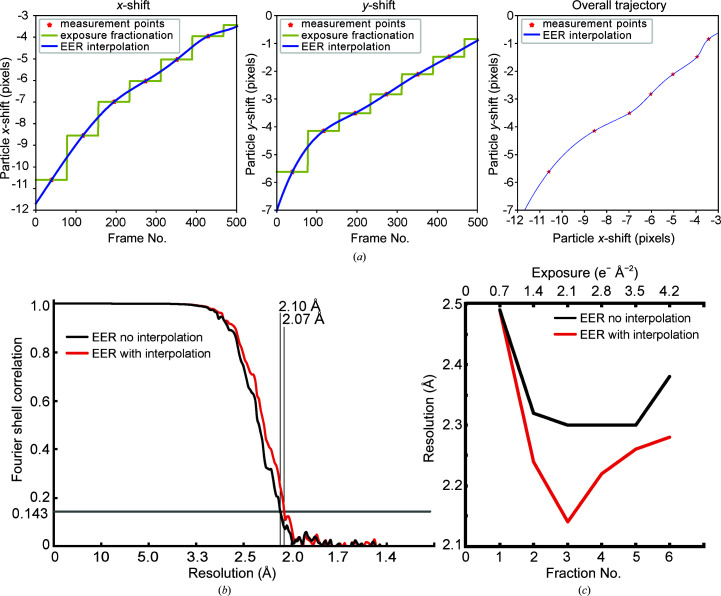
Improved correction of beam-induced motion with EER files. (*a*) Example of individual particle trajectories measured from 30 exposure fractions and interpolated to the physical frame rate of the camera. The yellow line represents the applied motion without the *B*-spline interpolation enabled by the EER method, while the blue line represents the interpolated trajectory enabled by EER. (*b*) Fourier shell correlation curve for 3D reconstructions without (black curve; 2.10 Å resolution at FSC = 0.143) and with (red curve; 2.07 Å resolution at FSC = 0.143) interpolated motion applied to the individual camera frames. (*c*) Comparison of resolution for 3D maps (FSC = 0.143) calculated from different exposure fractions, each corresponding to 0.7 e^−^ Å^−2^, without (black curve) and with (red curve) interpolated motion applied to the camera frames.

Using the implementation of the *alignparts_lmbfgs* algorithm (Rubinstein & Brubaker, 2015 ▸) in *cryoSPARC* (Punjani *et al.*, 2017 ▸), we measured the motion trajectory of 291 408 single-particle images of apoferritin. These trajectories were measured in EER movies that had been divided into 30 exposure fractions, where each exposure fraction was comprised of 77 camera frames. Images were recorded with a calibrated physical pixel size of 1.06 Å but supersampled 1.5 × 1.5 to super-resolution pixels of 0.7067 Å using information from the EER data. To mimic conventional movie processing, the motion measured from the 30 exposure fractions was applied uniformly to all of the frames within each fraction [Fig. 3 ▸(*a*), yellow line]. Exposure weighting, as proposed previously (Baker *et al.*, 2010 ▸), was performed as described in the *alignparts_lmbfgs* algorithm (Rubinstein & Brubaker, 2015 ▸) but using resolution-dependent optimal exposures that were measured subsequently (Grant & Grigorieff, 2015 ▸). This strategy is equivalent to the exposure weighting performed with *MotionCor*2 (Zheng *et al.*, 2017 ▸), *Unblur* (Grant & Grigorieff, 2015 ▸) and *cryoSPARC* (Punjani *et al.*, 2017 ▸). To assess the benefit of increased time resolution in the applied motion trajectories, third-order *B*-spline interpolation was used to assign the position of each particle in each camera frame [Fig. 3 ▸(*a*), blue line]. Three-dimensional reconstruction using just the measured motion from the 30 exposure fractions without interpolation produced a map at 2.10 Å resolution [Fig. 3 ▸(*b*), black curve]. In contrast, applying interpolated motion at the physical frame rate prior to averaging gave a map at 2.07 Å resolution, which is an improvement of two bins in Fourier space [Fig. 3 ▸(*b*), red curve]. Beam-induced motion in the early frames of a movie is thought to be one of the primary limits to resolution in cryoEM at present (Henderson, 2018 ▸). This modest improvement in resolution from interpolated application of the measured motion suggests that inaccuracy in the motion estimates may be limiting the extraction of information from finely fractionated exposures.

In contrast to the small improvement in resolution for the map calculated from all exposure fractions, the resolutions of 3D maps calculated from individual exposure fractions improved markedly when motion trajectories were interpolated and applied directly to camera frames. Movies with each fraction consisting of 77 frames, with 1.4 e^−^ per Å^2^ per fraction, were fractionated further to averages of 38 frames, corresponding to 0.7 e^−^ per Å^2^ per fraction. 3D maps were calculated separately from the first six of these new fractions with or without the application of the motion to the individual camera frames in each fraction. During this 3D reconstruction the orientations of the particle images were not changed from those measured from the exposure-weighted average of fractions. The resolutions of the resulting maps are shown in Fig. 3 ▸(*c*). Remarkably, the resolutions of these maps are only 0.07–0.4 Å worse than the resolutions of the maps calculated from the exposure-weighted average of all frames from the movies. This result indicates that while information from the entire exposure may guide the alignment of particle images to a 3D reference, the high-resolution features in maps can be reconstructed from just the earliest part of the exposure. While the first fraction is no better with the interpolated motion than with the non-interpolated motion, maps calculated from subsequent fractions show a marked improvement in resolution. Consequently, it appears that the estimated motion is not correct during the earliest part of the exposure where the specimen moves the most and with the least predicable direction. However, later in the exposure the estimated motion is sufficiently accurate to allow improved map resolution when the trajectory is interpolated and applied directly to the camera frames.

## 4. Discussion   

Processing of the EER images in this work required an intermediate image-processing step of converting EER data into a movie format that could be used by *cryoSPARC* (Punjani *et al.*, 2017 ▸) and *RELION* (Scheres, 2012 ▸), the software packages that we employed for image analysis. However, information about the EER file format has already been shared with the development teams for these software packages and the capability to directly read EER data has been implemented in both packages. The file-format specification is also available to other software developers.

DDDs have previously allowed the extraction of information beyond the physical Nyquist frequency of the camera for images of 2D crystals (Chiu *et al.*, 2015 ▸) and single particles (Feathers *et al.*, 2019 ▸), and other algorithms have been proposed to explore this approach further (Chen, 2018 ▸). When subdividing each physical pixel into 4 × 4 subpixels, the EER format allows the preservation of super-resolution information with an additional four bits required for each electron detected, which increases file sizes by a maximum of 57%. In contrast, conventional representations of a super-resolution image with each physical pixel divided into 2 × 2 subpixels causes a 400% increase in file size relative to the non-super-resolution image. Dividing the physical pixel into 4 × 4 subpixels, as performed in the EER format, would increase the file size by 1600%. Acquiring images at a lower magnification provides more particles per image and decreases the time spent preparing for exposure. However, super-resolution imaging does not provide a dramatically faster route to high-resolution cryoEM data collection. Decreasing the microscope magnification requires keeping the camera exposure rate (e^−^ per pixel per second) constant to allow electron counting and requires more time to obtain the same total specimen exposure (e^−^ Å^−2^). Nonetheless, the preservation of super-resolution information decreases the importance of the magnification chosen when data collection is initiated. Furthermore, a lower magnification increases the field of view in images, which can facilitate the measurement of specimen tilt and the microscope contrast-transfer function. A larger field of view may also improve the modelling of beam-induced motion, which typically utilizes information from the movement of adjacent particles (Scheres, 2014 ▸; Rubinstein & Brubaker, 2015 ▸). The increased field of view can also be advantageous for electron tomography of larger objects.

The calculation of 3D maps from different exposure fractions described in Fig. 3 ▸(*c*) shows that it is possible to obtain the highest resolution from a single exposure fraction after pre-exposure of the specimen with 1.4 e^−^ Å^−2^. This finding is consistent with the large body of evidence that the earliest part of the exposure, in which the high-resolution information should be best preserved, suffers from the most beam-induced specimen movement (Henderson, 2018 ▸). The position of this optimum indicates that smoother application of the measured particle motion from interpolation has the greatest effect near the beginning of the movie where motion is still large, while in the first 1.4 e^−^ Å^−2^ of exposure inaccuracies in the measured motion prevent the smoother application from improving the map resolution. This result is particularly encouraging. It suggests that new techniques that are capable of more accurate measurement of beam-induced motion could allow the extraction of high-resolution information from the earliest frames of a movie. EER data, which preserve the full temporal resolution of data acquired with DDD cameras while maintaining manageable file sizes, can allow the development of these improved beam-induced motion-correction methods.

## Supplementary Material

EMDB reference: apoferritin from super-resolution experiment with no supersampling, EMD-22346

EMDB reference: from super-resolution experiment with random sub-pixel values, EMD-22347

EMDB reference: from super-resolution experiment with EER sub-pixel values, EMD-22348

EMDB reference: from 1.06 Å per pixel dataset without supersampling, EMD-22349

EMDB reference: from intra-fraction motion correction experiment with no B-spline interpolation, EMD-22350

EMDB reference: from intra-fraction motion correction experiment with B-spline interpolation, EMD-22351
